# Assessment of a flow cytometry technique for studying signaling pathways in platelets: Monitoring of VASP phosphorylation in clinical samples

**DOI:** 10.1016/j.plabm.2018.02.002

**Published:** 2018-02-16

**Authors:** N. Mallouk, J. Varvat, A. Berger, M. Epinat, S. Accassat, A. Garcin, A. Montmartin, G. Li, P. Garnier, P. Mismetti, C. Lambert

**Affiliations:** a Unité de Médecine Vasculaire et Thérapeutiques, CHU Saint-Etienne, Hôpital Nord, Saint-Etienne, France; b Centre de Microscopie Electronique Stéphanois (CMES), Université Jean Monnet, Saint-Etienne, France; c Unité Neuro-vasculaire CHU Saint-Etienne, Hôpital Nord, Saint-Etienne, France; d Laboratoire d’Immunologie Clinique CHU Saint-Etienne, Hôpital Nord, Saint-Etienne, France; e Unité de Recherche Clinique Innovation et Pharmacologie (URCIP), CHU Saint-Etienne, Hôpital Nord, Saint-Etienne, France; f SAnté INgéniérie BIOlogie St-Etienne (SAINBIOSE), Université Jean Monnet, Saint-Etienne, France; g Service d’Urologie, CHU Saint-Etienne, Hôpital Nord, Saint-Etienne, France; h CNRS-UMR 5148-Ecole Nationale Supérieure des Mines de St Etienne (ENSM), Saint-Etienne, France; i GIMAP EA3064, Saint-Etienne, France

**Keywords:** Platelet signaling, VASP, Flow cytometry, Clopidogrel

## Abstract

A recently released kit (PerFix EXPOSE) was reported to improve the measurement of the degree of phosphorylation of proteins in leukocytes by flow cytometry. We tested its adaptation for platelets to monitor vasodilator-stimulated phosphoprotein (VASP) phosphorylation, which is the basis of a currently used test for the assessment of the pharmacological response to P2Y12 antagonists (PLT VASP/P2Y12). The PerFix EXPOSE kit was compared to the PLT VASP/P2Y12 kit by using blood samples drawn at 24 h post clopidogrel dose from 19 patients hospitalized for a non-cardio-embolic ischemic stroke and treated with clopidogrel monotherapy for at least five days in an observational study. The platelet PerFix method was based on adaptation of the volume of the sample, the centrifugation speed and the incubation temperature. Poor agreement between prevention by adenosine diphosphate (ADP) of PGE_1_-induced cAMP-mediated VASP phosphorylation and ADP induced aggregation assessed by Light Transmittance Aggregometry was found. We found a significant correlation between the PLT VASP/P2Y12 kit and the PerFix EXPOSE kit. The PerFix EXPOSE kit may also be helpful to monitor adverse effects of second-generation tyrosine kinase inhibitors on platelets.

## 1 Background

Platelet aggregation is a key parameter in arterial thrombosis, leading to myocardial infarction or ischemic stroke. Platelets have several receptors sensitive to different stimuli and drugs that modulate aggregation with potential critical clinico-pathological significance. Platelet intracellular signaling is essential for platelet function but difficult to study because of the small size and the need for delicate manipulation of platelets and because the reference techniques require large cell volumes and sample preparation. Flow cytometry is often used [1] in routine clinical laboratories.

Only one kit (PLT VASP/P2Y12) is available to clinicians and researchers to study phosphorylated vasodilator-stimulated phosphoprotein (pVASP) in platelets. This kit is limited to adenosine-di-phosphate (ADP) activation. Nevertheless, many other signaling pathways are involved in platelet pathophysiology and cannot be investigated with this kit. A new kit – PerFix EXPOSE – was recently released to measure by flow cytometry the degree of phosphorylation of various proteins in large cells as leukocytes and cell lines [2], [3]. It was developed to enhance the signal-to-noise ratio of most intra-cellular phospho-epitopes stainings and to simplify the steps necessary for the sample preparation. This kit was not initially intended for platelets studies, but the manufacturer has observed that platelets are preserved by the preparation procedure, which only lyses red blood cells. We decided to adapt this simple and robust method to our clinical requirement: studying platelet function directly in whole blood of patients treated with clopidogrel.

Clopidogrel is routinely used as a part of the current practice guidelines [4] for preventing arterial thrombosis recurrence. However, a significant fraction of patients (15.9–49.5%, depending on the assay used) show a low response to clopidogrel according to laboratory tests [5]. These patients exhibit a higher recurrence rate of vascular thrombosis [6], [7], [8].

The pharmacological target of clopidogrel is the platelet P2Y12 receptor, where it acts as an irreversible antagonist. An inhibitory pathway linking P2Y12, Gi, adenylate cyclase and pVASP is involved [1], [9], [10]: ADP activates the P2Y12 receptor and prevents VASP phosphorylation. Clopidogrel inhibits the effect of ADP by blocking the P2Y12 receptor, so VASP is phosphorylated. The kit PLT VASP/P2Y12 was developed to evaluate the pharmacological effect of clopidogrel on platelets by measuring the level of VASP phosphorylation.

In the test conditions, prostaglandin E_1_ (PGE_1_) induces maximal VASP phosphorylation. ADP activates P2Y12 receptor and prevents VASP phosphorylation. ADP has no stimulating effect on the P2Y12 receptor if it is inhibited by clopidogrel.

Flow cytometry tests that can analyze individual cells use whole blood. The PerFix EXPOSE kit should be assessed and compared to the current laboratory tests in simple conditions before further investigation. In this study, we evaluated the adaptation of the PerFix EXPOSE kit to platelets by taking VASP phosphorylation as an example because it has been extensively used in clinical trials and we used an existing ongoing observational study to study how results from the two kits agree.

## 2 Materials and methods

The degree of platelet VASP phosphorylation was assessed on patients hospitalized for a non-cardio-embolic ischemic stroke, included in an observational study entitled “Evaluation of the Biological Response to Clopidogrel in Patients with Ischemic Stroke.” or AAPIX, as referred in clinicalTrials.gov with the identifier NCT01955642 in the Stroke Center of Saint-Etienne Hospital.

Patients (12 men, 7 women, mean age: 72 years; inclusion between November 2014 and September 2015) were treated orally with a regular daily dose of 75 mg of clopidogrel (Plavix 75 mg, Sanofi Pharma, Bristol-Myers Squibb SNC, Paris, France) for at least 5 days to be at steady state, before blood withdrawal. Patients treated with aspirin were excluded. All patients were informed of the study objectives and gave their consent to participate in the protocol. The study was performed in accordance with the principles of the Declaration of Helsinki and was approved by the local Ethics Committee (Rhône-Alpes, Loire) and French health regulations. Blood samples from healthy volunteers were a kind gift from the Blood Transfusion Center (EFS Auvergne-Loire; Saint-Etienne, France).

Sample preparation was based on recommended procedures for platelet aggregation and flow cytometric assays [11], [12], [15]. Briefly, blood was drawn with a 19-gauge needle and the first 2.5 ml was discarded. All samples were collected in citrated tubes (Sodium Citrate 0.105 M/3.2%, Becton Dickinson, Plymouth, UK), before oral administration of clopidogrel – i.e. 24 h after the previous dose. The tubes were gently mixed by two inversions immediately after blood draw.

The VASP assay was performed according to the manufacturer instructions by using the PLT VASP/P2Y12 kit (Diagnostica Stago, Asnières, France). In brief, platelets from whole blood were treated with PGE_1_ as reference value, from which the prevention of cAMP-mediated VASP phosphorylation induced by ADP was measured. Samples were incubated for 10 min at room temperature and the reaction was stopped by cell fixation for 5 min. PGE_1_ and ADP concentrations were not stated in the manufacturer's literature. Platelets were then permeabilized and labeled at room temperature using an antibody antipVASP (Ser239) mouse monoclonal or a negative isotypic control (mouse monoclonal antibody) for 5 min before adding a polyclonal anti-mouse IgG-FITC (fluoresceine isothiocyanate) and a platelet counter-staining reagent-PE, antibody antiCD61-PE (phycoerythrin) for 5 min. Then 2 ml of final diluting buffer was added prior to immediate acquisition.

In addition, our Platelet PerFix flow cytometry method was developed from the PerFix EXPOSE kit provided for leukocytes (Beckman-Coulter; Brea, CA, USA). We adapted the manufacturer's instruction for this kit. The fixation, permeabilization, staining and washing reagents of the kit were used. Each step was performed at room temperature.

Whole blood was incubated with PGE_1_ or PGE_1_ with ADP as detailed in Table 1. The reagents 2a and 2b from the PLT VASP/P2Y12 kit were used. The samples were fixed for 10 min then permeabilized for 5 min. Red blood cells were also lysed at this step. The samples were centrifuged (1200 *g*, 2–3 min) and the supernatant was discarded. The pellet was suspended in the staining reagent and stained with an antibody antiCD61-PE-Cy7 (phycoerythrin-cyanine 7, clone SZ21, Beckman Coulter) and an antibody antiSer-239 pVASP-FITC 0.4 µl (clone 16C2, Nanotools, the same clone as used in the PLT VASP/P2Y12 kit) or an antibody antiCD45-FITC (clone J33, Beckman Coulter) as isotype control for 20 min in the dark. pVASP labeling was determined by a titration method performed with platelets treated with PGE_1_ from healthy volunteers. After the addition of 3 ml of the washing reagent provided (Phosphate Buffered Saline, formaldehyde and detergent) and centrifugation, the platelets were suspended in 500 µl of the washing reagent. In brief, our adaptation led to a ten-fold reduction of the volume of whole blood and reagents (fixation, permeabilization), and to a higher speed and a shorter time of centrifugation as compared to the manufacturer instructions for leukocytes. An antibody antiPhospho-GSK-3alpha (Ser21) (Cell Signaling Technology) followed by an antibody antiCD61-PE and a polyclonal antibody antimouse IgG-FITC (fluoresceine isothiocyanate) have also been used by indirect fluorescence.

**Table 1:** Adaptation of the PerFix EXPOSE kit for platelets

**Kits**	**PerFix EXPOSE : protocole for platelets**	**PLT VASP/P2Y12 kit**	**Strategy**
**Sample set up**	On a rack, per sample, setup 3 plastic tubes labeled T1, T2 and T2c	On a rack, per sample, setup 3 plastic tubes labeled T1, T2 and T3:	The idea was to spare and reduce the volume (10 µl) of blood as in the PLT VASP/P2Y12 kit
	In tubes T1, T2 and T2c : pipette **10 μL** of whole blood.	In tube T1 : pipette **10 μL** of PGE1.	Whole bood was chosen to spare time
		In tubes T2 and T3 : pipette **10 μL** of PGE1+ADP.	
**Activation**	PGE1 (reagent 2a) and PGE1 with ADP (reagents 2b) **from the PLT VASP/P2Y12 kit**	In tubes T1, T2 and T3 : pipette **10 μL** of whole blood.	PGE1 (reagent 2a) and PGE1 + ADP (reagents 2b) from the PLT VASP/P2Y12 kit were used to compare the degree of phosphorylation of VASP between the kit PerFix EXPOSE and the kit PLT VASP/P2Y12.
	In tube T1 : pipette **10 µl** of PGE1	Homogenize the tubes for 1–2 s using a vortex set on low speed.	It wasn't necessary to incubate at 37 °C
	In tubes T2 et T2c : pipette **10 µl** PGE1 et ADP	Incubate for **10 min** at **room temperature**.	
	Homogenize the tubes for 1 to 2 s using a vortex set on low speed.		
	Incubate **for 10 min** at **room temperature**.		
**Fixation**	In tubes T1, T2 and T2c : pipette **5 μL** of fixative reagent	In tubes T1, T2 and T3 : pipette **10 μL** of reagent 3.	The volume of the fixative reagent was adapted to the volume of the sample used
	vortex immediately using vortex set on low speed (1-2 seconds)	Homogenize the tubes for 1–2 s using a vortex set on low speed.	
	Incubate the tubes at **RT** for **10 min**.	Incubate the tubes at **room temperature** for **5 min**.	
**Permeabilization**	**100 µl** of permeabilizing reagent **at RT** for **5 min**	**Cell permeabilization and immunolabeling**	The volume of the permeabilizing reagent was adapted to the volume of the sample used
		In tubes T1 and T2 : pipette **10 μL** of reagent 4a.	
		In tube T3 : pipette **10 μL** of reagent 4b.	Platelets were unaffected by the procedure used for red blood cells lysis with preservation of the membrane protein used to identify platelets
		Homogenize the tubes for 1–2 s using a vortex set on low speed.	
		Incubate the tubes **at room temperature** for **5 min**.	It wasn't necessary to incubate at 37 °C
**Wash step**	Centrifugation à 1200 *g* pendant 2-3 min **at RT**		The centrifugugation at 1200 *g* for a short duration did not alter platelets
	Discard the supernatant		
**Fluorescent staining**	Resuspend the pellet in **100 µ**l of staining reagent	Fluorescent staining and platelet counter-staining	Same procedure as the PerFix EXPOSE instructions for leucocytes
	T1 et T2 : 10 µl of antibody anti CD61-PC7 and 0.4 µl of antibody anti pVASP-FITC	In tubes T1, T2 and T3 : pipette 10 μL of reagent 5.	Antibody anti CD61 and anti Ser-239 pVASP(clone 16C2) already present in the PLT VASP/P2Y12 were chosen to compare the degree of phosphorylation of VASP between the kit PerFix EXPOSE and the kit PLT VASP/P2Y12.
	T2c : 10 µl of antibody anti CD61-PC7	Homogenize the tubes using a vortex for 1–2 s set on low speed.	
	Incubate for **20–25 min** at RT **protected from light**	Incubate the tubes at room temperature for **5 min**.	Conjugated fluorochrome was used for direct immunolabeling
**Wash step**	Add **3 ml** of washing reagent in each tube.	Add **2 mL** of reagent 1 in each of the 3 tubes.	The centrifugugation at 1200 *g* for a short duration did not alter platelets
	Centrifuge at **1200 *g*** for **2-3 min** at RT	Homogenize the tubes for 1–2 s using a vortex set on high speed	
	Discard the supernatant		
	Resuspend the platelets in **500 µl** of washing reagent in each tube. analysis on a flow cytometer.	The contents of the tubes may be stored for **2 h at 2–8 °C** before platelet analysis.	
**Time of the procedure**	60 min		

RT=room temperature.

Platelets were analyzed on a Navios cytometer using Navios software (Beckman-Coulter) within 1 h after the preparation was completed. FlowCheck Pro Fluorospheres (Beckman-Coulter) were used for daily verification of the Navios cytometer optical alignment and fluidics system. FlowSet Pro Fluorospheres (Beckman-Coulter) were used to calibrate and standardize the fluorescence detectors. Settings were optimized and fluorescence overlap compensation calculated using single labeling, isotype controls and Full Minus One (FMO) procedure, on PGE_1_ treated platelets from healthy donors [13]. Acquisition was performed on 10,000 events. Data were then analyzed by using WinMDI Analysis Software, version 2.8. The phosphoVASP-FITC MFI (Mean Fluorescence Intensity) was determined on CD61 positive platelets. Our cytometric results were expressed in MFI. As recommended by the PLT VASP/P2Y12 kit manufacturer, PRI (Platelet Reactivity Index) was calculated according to the following formula:

$$PRI=\left[\left(MFIc\;PGE_{1}–MFIc\;\left(PGE_{1}+ADP\right)\right)/MFIc\;PGE_{1}\right]x100$$

based on the corrected Mean Fluorescence Intensity (MFIc) defined as:

$${\mathrm{MFIc}=\mathrm{MFI}}_{\left(\mathrm{PGE1}+\mathrm{ADP}\right)}{–\mathrm{MFI}}_{\left(\mathrm{isotype control}\right)}$$

PRI should be strongly lowered by clopidogrel treatment. According to the literature, clopidogrel response according to laboratory tests is considered as low if the PRI value is > 50% [5]. We accepted the same commonly accepted PRI cut-off value for our Platelet PerFix assay.

Maximum platelet aggregation induced by ADP was measured by Light Transmittance Aggregometry or LTA (TA_4V aggregometer; SD-Medical, Heillecourt, France). LTA is used routinely, according to the manufacturer's instructions and recommendations of the platelet physiology subcommittee of SSC/ISTH [14], [15]. The ADP optimal concentration inducing a maximum platelet aggregation was determined at 10 μM [16]. Platelet-rich plasma was prepared from citrated blood (Sodium Citrate 0.105 M/3.2%) by centrifugation (150 *g*, 10 min). The adjustment for platelet count was not performed, as recommended [15].

According to the literature [5], clopidogrel response according to laboratory tests is usually considered as low if a maximal platelet aggregation value is > 70% [5].

The mean values of measurements, the linear regression and the coefficient of determination were calculated with Excel 2010 (Microsoft, Redmond, WA, USA). The values were expressed as the mean +/- standard deviation (SD); the coefficient of variation (CV) was defined as (SD/mean) * 100.

## 3 Results

### 3.1 Evaluation of the degree of phosphorylation of platelet VASP (kit PLT VASP/P2Y12)

**Fig. 1:**
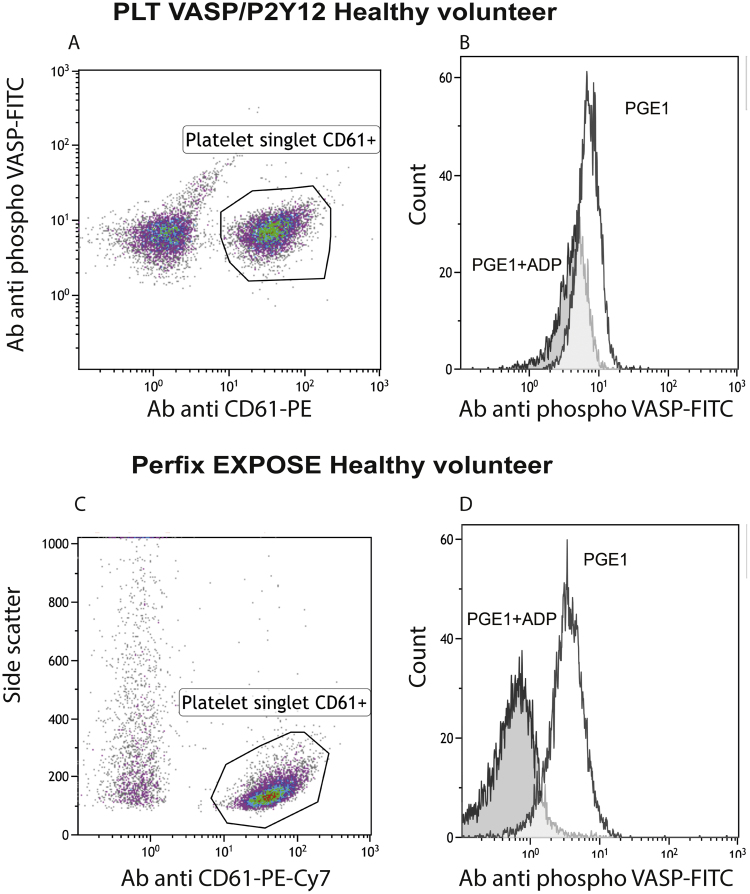
Gates used for the analysis of platelets identified as CD61 + population (A–C). Whole blood samples were drawn from healthy volunteers. Representative histograms for PGE_1_ (white) versus PGE_1_ + ADP conditions (gray, B, D). PLT VASP/P2Y12 kit (A–B) and the PerFix EXPOSE kit (C-D). Ab = antibody.

As recommended by the manufacturer, PGE_1_ is added to induce the full phosphorylation of VASP, then, ADP is added in the test tube to measure the prevention of cAMP-mediated VASP phosphorylation. Gates used for the analysis of platelets identified as CD61 + population are shown in Fig. 1(A and C).

In a representative healthy control, the PGE_1_ induced maximized platelet phosphorylation of VASP with a MFI value (PGE_1_) of 7. This was lowered by ADP to a MFI value (PGE_1_ + ADP) of 4.1 (Fig. 1b) while the non-specific fluorescence MFI value (isotype control) was 3.9, generating a PRI = 95.2%. Representative overlaid histograms of PGE_1_ (white) and PGE_1_ + ADP (gray) are shown in Fig. 1B.

On platelets from 7 healthy donors, the mean PRI value was 91.8% +/− 3.9, CV = 4.2% (data not shown).

### 3.2 Our Platelet PerFix method

Red blood cells lysis did not alter membrane protein used to identify platelets, ADP and PGE1 effects on platelets were preserved. For platelets from 7 healthy donors, the mean PRI value was 82.7% +/− 7.2 (CV = 8.7%; data not shown). For a sample tested 4 times with the same kit, mean MFI (PGE_1_) was 23.3 +/− 1.7 (CV = 7.4%). The gate used for the analysis of platelets identified as CD61 + population is shown in Fig. 1C. The numerous events in the left upper part could be leucocyte debris but not platelets (CD61 negative).

The platelet VASP phosphorylation maximized with PGE_1_ was detected in a representative healthy control with a MFI value (PGE_1_) at 3.4. This signal was clearly separated from the background that could be measured either on the debris or with the isotype control. ADP induced a decrease of VASP phosphorylation with a MFI value (PGE1 + ADP) at 0.5 while the corresponding MFI value (control) was 0.2 generating a PRI value of 90.3%. Representative overlaid histograms of PGE_1_ (white) and PGE_1_ + ADP (gray) are shown in Fig. 1D.

### 3.3 Comparison of our adapted Platelet PerFix method with the PLT VASP/P2Y12

**Fig. 2:**
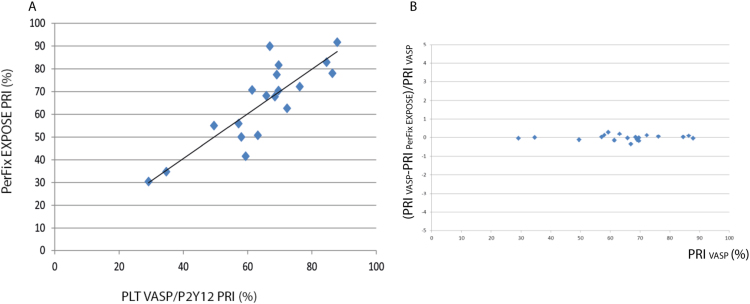
Correlation between PRI values obtained with the PLT VASP/P2Y12 and the PerFix EXPOSE kits (A). R2 = 0.7, slope = 0.98 (n = 19). Bland – Altman plot (B).

The PRI values obtained with our adapted test were directly correlated to the values obtained with PLT VASP/P2Y12 in 19 patients, giving a linear regression (slope = 0.98, coefficient of determination r^2^ = 0.7 as illustrated in Fig. 2). A Bland – Altman plot is shown in Fig. 2B.

The Platelet PerFix could clearly differentiate a good clopidogrel inhibition of ADP induced activation. Overall, in 19 patients, taking a cut-off PRI value of 50% in the two cytometry assays, 2 samples were measured as good responders and 15 samples as poor responders in agreement between the two assays; 15 samples were poor responders with our test out of 16 poor responders as detected by PLT VASP/P2Y12 giving a sensitivity of 93.75%. The number of good responders was too low to reliably calculate the specificity. The PRI values of two samples were around the cut-off values: one had a PRI value of 55% on our test as compared to 49.5% with the PLT VASP/P2Y12 assay and a maximal platelet aggregation value of 69.5%. The other one had PRI values of 41.6% and 59.4% respectively, and a maximal platelet aggregation value of 69.8%.

### 3.4 Comparison of our adapted Platelet PerFix method with Light Transmission Aggregation (LTA) as the reference method

**Fig. 3:**
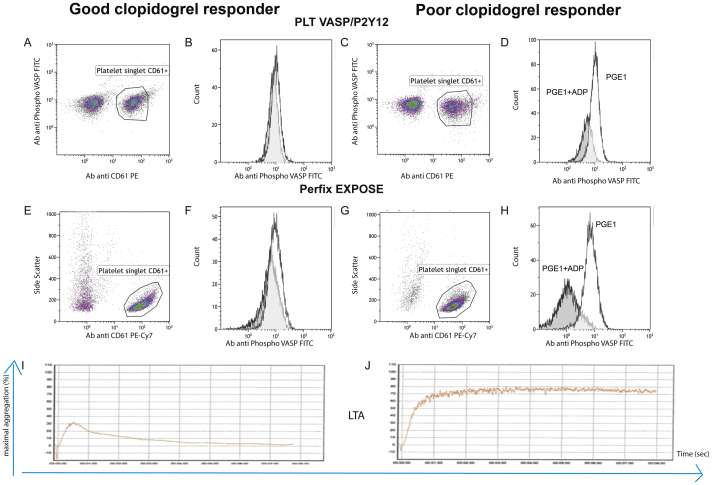
Illustration of good (A, B, E, F and I) and poor clopidogrel response profiles (C, D, G, H and J). Gates used for the analysis of platelets identified as CD61 + population (A-C-E-G). Representative histograms for PGE_1_ (white) versus PGE_1_ + ADP conditions (gray, B, D, F and H). PLT VASP/P2Y12 (A–D) versus PerFix EXPOSE kits (E–H). Platelet aggregation was induced by ADP 10 μM (I–J). Maximum platelet aggregation was measured.

Representative LTA results from a good and a poor responder to clopidogrel gave maximal platelet aggregation values of 31.3% (Fig. 3I) and 77.5% (Fig. 3J), PRI values were 30.5% and 82.9% with our Platelet PerFix test and 29.2% and 84.6% with the PLT VASP/P2Y12 assay.

The gate used for the analysis of platelets identified as CD61 + population is shown in Fig. 3A and C (PLT VASP/P2Y12, good and poor clopidogrel responder according to laboratory tests) and in Fig. 3E and G (PerFix EXPOSE, good and poor clopidogrel responder). Events outside of the platelet gate in Fig. 3A were platelet aggregates.

Representative overlaid histograms of PGE_1_ (white) and PGE_1_ + ADP (gray) are shown in Fig. 3B and D (PLT VASP/P2Y12 assay, good and poor clopidogrel responder) and in Fig. 3F and H (Platelet PerFix test, good and poor clopidogrel responder).

Overall, in our 19 patients, with a cut-off value of 50% for our PerFix EXPOSE test and 70% for the LTA assay, 3 patients were measured as good responders and 8 as poor responders in agreement between the two assays (sensitivity 100%). On the other hand, out of the 11 patients classified as good responders on LTA, only 3 were found as good responders on Platelet PerFix test (specificity: 27.3%) but the same result was found with the PLT VASP/P2Y12 assay. Among the 8 patients considered as poor responder in flow cytometric assays (PRI value > 50%) 4 had a maximal platelet aggregation value between 65% and 70% and 4 had a maximal platelet aggregation value between 43% and 55%. The results obtained by comparing the PLT VASP/P2Y12 with the LTA assay were in the same range.

**Fig. 4:**
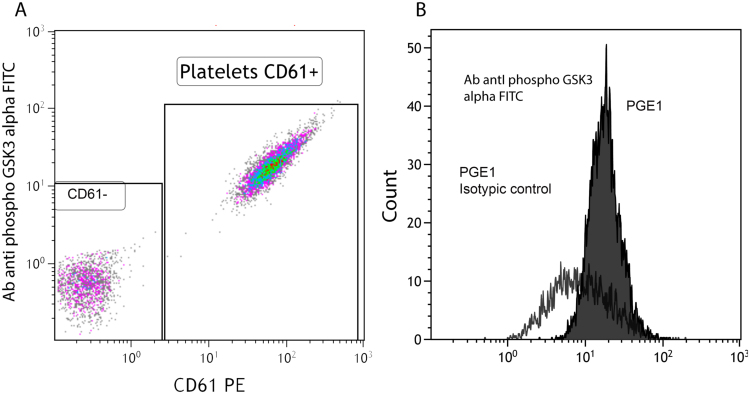
The Gate used for the analysis of platelets was identified as CD61 + population (A). Whole blood sample was drawn from a healthy volunteer. Representative histograms for PGE_1_ with the PerFix EXPOSE kit (B). CD61- = CD61 negative population;.

Our method is open for assessing other signaling pathways. An example of this potential flexibility is given with an antibody antiphospho-GSK-3alpha (Ser21) (Fig. 4). Similarly, platelets selected on CD61 expression showed a GSK3 alpha labeling compared to isotype control.

## 4 Discussion

From the technical perspective, measuring signal transduction in platelets is difficult because of their fragility, the impact of the pre-analytical sample conditions, their small size and the requirement of a low signal/noise ratio. Taking advantage of a new kit dedicated to signaling studies in leukocytes, we adapted the simplified method for platelets by using the same optimized reagents. Red blood cell lysis did not alter the level of antigen expression nor the cAMP mediated VASP phosphorylation.

We removed the 37 °C incubation steps (all steps were performed at room temperature), shortened the centrifugation step of fixed platelets, and we decreased the volumes of reagents by up to 10-fold.

This method was applied directly to whole blood, avoiding artefacts due to the initial centrifugation steps frequently performed when “platelet-rich plasma” is required. This also saved time and effort.

In order to validate this approach, we compared our Platelet PerFix method with the well-accepted PLT VASP/P2Y12 method in measuring Ser239 pVASP for monitoring anti-aggregant therapy. The reference method is not really a gold standard, but it should be the most appropriate test available since this assay has been correlated with the plasma concentration of the active metabolite of clopidogrel [17]. Both methods required small volumes of whole blood (10 µl per tube). The PerFix EXPOSE method takes longer than the kit PLT VASP/P2Y12 (1 h) but is open to the use of a large panel of antibodies whereas the PLT VASP/P2Y12 is limited to this specific application with pVASP antibody with ADP as agonist.

For the comparison of PLT VASP/P2Y12 and the PerFix EXPOSE kit adapted to platelets, PGE_1_ and ADP reagents from the PLT VASP/P2Y12 assay were used. Although PGE_1_ and ADP concentrations were not detailed in the manufacturer's instructions for commercial reasons, typical values can be found in the literature [1], [18], [19].

Interestingly, we observed a much wider dynamic range with our procedure, with a strong signal of phosphoVASP and a low background which can be explained by less non-specific binding. Buffers were enriched with BSA and salts and detergents were optimized for the immunolabeling. This confirms that this kit detects phosphoepitopes, even in platelets.

Comparison of our test with the PLT VASP/P2Y12 assay has shown very good agreement. Using the same cut-off value defined for the PLT VASP/P2Y12 assay, only 2 patients showed discordant values, probably because of the lack of precision.

Unfortunately, the proportion of good clopidogrel responders was too low to evaluate the specificity of the PerFix EXPOSE test. This was due to the specific conditions of the AAPIX study. New patients, after a 600 mg loading dose of clopidogrel or on maintenance therapy with prasugrel or ticagrelor should be tested to detect a higher proportion of clopidogrel responders.

From the clinical perspective, we have included a small group of ischemic stroke patients. They were treated with second line clopidogrel treatment because they had experienced a previous ischemic stroke under aspirin treatment. Our group contained many low responders (16/19), only 1 good responder, and 2 cases around the cut-off value (where the only discrepancy between the kits was observed). It is worth noting that the literature concerning poor clopidogrel response for ischemic stroke patients is scarce. However our results are in line with previous studies showing a high prevalence (44–50%) of poor responders in ischemic stroke patients, according to the assay used [20], [21], [22], when compared to patients with coronary heart disease [5], [7]. Nevertheless, our value exceeded previous results. This could be due to the low statistical power of our study and due to the fact that patients had not been included consecutively. But the same tendency was found in the global AAPIX study that involved many low responders. Several clinical factors can explain such a result: these patients were treated with a regular dose of clopidogrel (75 mg) and no other anti-aggregant (such as aspirin), as recommended in ischemic stroke thrombosis because of the high risk of bleeding [4]. Our patients were included at an early stage of the disease, most probably with an acute phase reaction that favors platelet activation. The cut-off values are probably not optimized even if they have been used for patients with coronary artery disease (PRI value of 50% and LTA of 70%). Appropriate cut-off values should be assessed for ischemic stroke patients in a larger study.

Interestingly the LTA assay also showed elevated values (8/19 low responders) compared to the literature [7]. Poor agreement between the VASP assays and LTA was found as previously reported. This is not surprising because different aspects of the pharmacodynamic effects of clopidogrel are involved. The VASP assays evaluate the degree of phosphorylation of intraplatelet VASP proteins while LTA assesses the aggregation of platelets and the two assays are still considered to be complementary [6]. Biological monitoring of clopidogrel is no longer used in cardiology since the publication of the ARTIC and ANTARCTIC trials [25], [26] but it remains helpful for patients treated with clopidogrel as second line treatment after an ischemic stroke especially in decision-making in difficult clinical cases [23].

The aim of our work was to test a new simplified method and VASP was chosen as a reference system. Our method is opento other antibodies and other signaling pathways. Results with an antibody antiphospho-GSK-3alpha (Ser21) are encouraging and are in accordance with the results of Spurgeon [18].

It would be now interesting to go further by studying more receptors or more agonists of interest. We showed that this PerFix EXPOSE kit could be used easily under routine clinical conditions.

Furthermore, besides the prevention of arterial thrombosis, more treatments are targeting protein phosphorylation through tyrosine kinase. This targeted therapy is applied generally in leukemia or solid tumors with good efficiency, but platelets are then exposed to the same drugs with possible side effects on platelet function. Second-generation tyrosine kinase inhibitors like dasatinib which also affect scr-family kinase alter platelet function and may induce bleeding events [24].

In conclusion, our method can detect low responders to clopidogrel. Even if we were unable to evaluate the specificity of our test because of the high proportion of low clopidogrel responders in the AAPIX study, our work is a first step in the assessment of this method which is still appropriate for the analysis of multiple signaling pathways.
